# Molecular Survey on *Toxoplasma* *gondii* and *Neospora* *caninum* Infection in Wild Birds of Prey Admitted to Recovery Centers in Northern Italy

**DOI:** 10.3390/microorganisms9040736

**Published:** 2021-04-01

**Authors:** Alessia Libera Gazzonis, Luca Villa, Emanuele Lubian, Sara Ressegotti, Guido Grilli, Stefano Raimondi, Sergio Aurelio Zanzani, Maria Teresa Manfredi

**Affiliations:** 1 Department of Veterinary Medicine, Università degli Studi di Milano, 26900 Lodi, Italy; luca.villa@unimi.it (L.V.); emanuele.lubian@hotmail.com (E.L.); sara.ressegotti@studenti.unimi.it (S.R.); guido.grilli@unimi.it (G.G.); sergio.zanzani@unimi.it (S.A.Z.); mariateresa.manfredi@unimi.it (M.T.M.); 2 CRFS LIPU “La Fagiana”, 20013 Pontevecchio di Magenta, Italy; 3 Associazione CRAS di Vanzago, 20010 Vanzago, Italy; boscovanzago@wwf.it

**Keywords:** protozoa, genotype, toxoplasmosis, neosporosis, raptor, Wildlife Recovery Centre

## Abstract

*Toxoplasma gondii* and *Neospora caninum* (Apicomplexa, Sarcocystidae) are protozoan parasites infecting a wide range of intermediate hosts worldwide, including birds. Raptors acquire the infections through the ingestion of both infected preys and oocysts in the environment suggesting they might be used as indicators of the spread of these pathogens. Here, we report an epidemiological survey with the aim of determining the prevalence of *T. gondii* and *N. caninum* infections in wild birds of prey, hospitalized in two Wildlife Recovery Centres (WRCs) in Northern Italy. Genomic DNA extracted from brain tissue samples was submitted to Real Time PCR targeting *T. gondii* B1 and *N. caninum* Nc5 genes. *T. gondii* genotyping was then performed by multilocus sequence typing (MLST) analysis, targeting three polymorphic genes (GRA6, BTUB, and altSAG2). *T. gondii* DNA was found in 35 (62.5%) out of 56 examined samples; concerning genotyping, it was possible to amplify at least one gene for 26 animals, and obtained sequences belonged to Type II. *N. caninum* DNA was only detected in two (3.6%) common kestrels (*Falco tinnunculus*), adding a new species to the list of suitable intermediate hosts for this pathogen. Data obtained in the present study thus confirmed the spread of both *T. gondii* *and N. caninum* in wild bird of prey, endorsing the role of WRCs in the epidemiological surveillance of wildlife.

## 1. Introduction

*Toxoplasma gondii* and *Neospora caninum* are two closely-related parasites belonging to the family Sarcocystidae (Apicomplexa). They have a worldwide distribution and are considered to be major abortigenic pathogens of domestic ruminants. *T. gondii*, which has the cat as a definite host, is able to infect a wide range of mammals and birds as intermediate hosts. *N. caninum*, although capable of infecting numerous species of mammals and birds, has bovine as its main intermediate host. Definitive hosts, such as dogs and wild canids, can become infected by ingesting the placentas or fetal cravings of infected intermediate hosts [1,2].

For both parasites, a sylvatic cycle is described as interfacing with the domestic cycle, which is often considered to be the primary cause of infection in livestock farms [3]. In fact, both pathogens were demonstrated by serological, histological, or molecular studies in several wildlife species comprising birds, as recently reviewed [4,5]. Among the avian species, birds of prey are of particular interest because, acquiring the infections mainly through the ingestion of infected preys (other birds, or small mammals), they can be used as sentinel species of environmental contamination with these parasites [4].

In raptors, various seroepidemiological and molecular studies reported *T. gondii* infection with different prevalence values, depending on the host species, the geographical area of origin, and the diagnostic technique used [6,7,8,9,10]. Raptors are considered resistant to clinical infection. However, clinical toxoplasmosis was described in a Canadian barred owl (*Strix varia*) that died following a collision with a car, in which severe hepatitis was found at autopsy [11]. Clinical toxoplasmosis was also observed in a bald eagle (*Haliaeetus leucocephalus*) found in a situation of distress and weakness that died of severe myocarditis [12]. Recently, another case of acute fatal toxoplasmosis was described in a juvenile bald eagle with concomitant severe emaciation and poxviral dermatitis [13].

*N. caninum* infection was reported in birds [5]; particularly, considering raptors, *N. caninum* DNA so far was detected, to the best of our knowledge, only in the brain tissue of a naturally infected common buzzard in Spain [9].

Investigating the prevalence of *T. gondii* and *N. caninum* in wildlife is necessary to understand the life cycle of the parasite, the dynamics of transmission, and the risk to public health [14]. Therefore, a molecular epidemiological survey was planned with the aim of determining the prevalence of *T. gondii* and *N. caninum* infections in wild birds of prey admitted to the Wildlife Recovery Centers (WRCs) located in the Lombardy region (Northern Italy). The study area is of particular interest as it is highly populated; there are also numerous farms dedicated to the breeding of both cattle and small ruminants. The sampled birds of prey, living in an area under such strong human and zootechnical pressure, are therefore perfectly placed at the interface between the domestic and sylvatic cycle of both *T. gondii* and *N. caninum*.

## 2. Materials and Methods

### 2.1. Study Population and Sample Collection

Sampling was performed between April 2018 and February 2019 in two WRCs in the Lombardy region (Northern Italy). WCR1 is located within the WWF (World Wildlife Fund) Natural Oasis of Vanzago (45°31′14.16″N 8°58′29.74″E) about 20 km from the metropolitan city of Milan. The number of animals hospitalized is constantly increasing, with about 500 raptors hospitalized in 2018. The LIPU (Lega Italiana Protezione Uccelli) “La Fagiana” WCR2 is located in the “La Fagiana” nature reserve of the Ticino Park (Pontevecchio di Magenta, Milan); although the number of hospitalizations per year is lower than that of WRC1 (about 300 birds of prey in 2018), Ticino Park represents an ideal environment for the release of rehabilitated specimens (45°26′13.2″N 8°50′18.6″E).

A total of 56 birds of prey (29 animals from WRC1 and 27 from WRC2), hospitalized and then died or euthanized, were recruited for the study. All animals included in the study died (by euthanasia or natural death) within the first days of hospitalization. During this period, the animals were kept in boxes inside the WRC infirmary, where access to other birds was not possible. Furthermore, all hospitalized raptors were fed defrosted food (chicks or mice), potentially containing non-infectious parasitic cysts. The short-stay time, the impossibility of access for prey animals, and the defrost diet thus ensured that the animals included in the study did not acquire *T. gondii* or *N. caninum* infection during their stay at the WRC. Brain samples were collected at the laboratories of the Department of Veterinary Medicine, Università degli Studi di Milano, during necropsy of wild birds performed within the West Nile Virus and the avian influenza virus epidemiological surveillance system conducted by the Local Health Authority. The brain was extracted after opening the skull; an aliquot of tissue (about 0.5–1 gr) was taken for the present study, mechanically homogenized with a sterile spatula, and placed in a single tube marked with a progressive identification number. Samples were stored at −20 °C until analysis.

Eleven species belonging to the Families Accipitridae (5), Falconidae (3), and Strigidae (3) were represented. All species included in the study are listed on the IUCN Red List of Threatened Species as “least concern” (https://www.iucnredlist.org/, accessed on 7 December 2019); only black kite (*Milvus migrans*) is listed as “near threatened” in the Italian Red List (http://www.iucn.it/liste-rosse-italiane.php, accessed on 10 February 2021).

Individual data concerning age was recorded or estimated on the basis of the size, weight, and plumage features, according to the species, classifying the animals as young or adult [15,16]. The reason for admission to the WRCs was also recorded. For each species, the dietary habits (mainly mammals, mainly birds, or generalist species) and the main migratory behavior (migrant or sedentary species) were registered [15,16].

### 2.2. Molecular Analysis

Tissue samples were processed for DNA extraction using a commercial kit (Nucleospin tissue, Macherey-Nagel GmbH and Co. KG, Düren, Germany), following manufacturer’s instruction. Extracted DNA was stored at −20 °C until analyzed.

For the detection of *T. gondii* DNA, samples were subjected to a real-time PCR (B1 real-time PCR), targeting a region of about 129 bp within the 35-fold repetitive B1 gene (AF179871) [17], as described by Gazzonis et al. [18], with slight modifications. For the detection of *N. caninum* DNA, a real-time PCR (Nc5 real-time PCR) targeting the Nc5 region was performed [19].

Both real-time PCRs were performed in a final volume of 20 μL, containing the PowerUp™ SYBR^®^ Green Master Mix (Thermo Fisher Scientific, Life Technologies, Monza, Italy) 2×, 0.5 μM of each primer [ToxB41f (5′-TCGAAGCTGAGATGCTCAAAGTC-3′) and ToxB169r (5′-AATCCACGTCTGGGAAGAACTC-3′) for *T. gondii*, NeoF (5′-ACTGGAGGCACGCTGAACAC-3′), and NeoR (5′- AACAATGCTTCGCAAGAGGAA-3′) for *N. caninum*], and 5 μL of DNA samples (approximately 250–500 ng of genomic DNA). Amplification and melting analysis were performed in a QuantStudio™ 3 Real-Time PCR System with a QuantStudio™ 3 software system (Applied Biosystems™ LSA28137), with the following cycling profile: incubation at 50 °C for 2 min, denaturation at 95 °C for 2 min, amplification for 40 cycles at 95 °C for 15 s, and 60 °C for 60 s, and a final step of melting analysis. Positive and negative controls (no template DNA) were included in each run; positive controls consisted of genomic DNA of *T. gondii* and *N. caninum*, previously amplified and identified by sequencing, extracted from the tissue samples of a coypu (lungs) [20] and a bovine fetus (pooled organs) [21], respectively. The melting program, consisting of temperature increases from 60 °C to 95 °C at intervals of 0.15 °C/s, was performed at the end of each cycle. Each sample was analyzed in duplicates, and the mean cycle threshold (Ct) and melting temperature (Tm) values were recorded. A sample was defined as positive when there was (i) a detectable amplification curve, (ii) a Ct value below 35, and (iii) a Tm value of ±0.5 °C vs. Tm value of positive control was recorded.

For *T. gondii* genotyping purposes, samples scoring positive to B1 real-time PCR were submitted to Multilocus sequence typing (MLST) analysis, targeting selected polymorphic genes (GRA6, BTUB, and altSAG2), following the nested PCR protocol described [22], with slight modifications. Unlike the described protocol, providing a multilocus PCR, the selected markers were each amplified in a different reaction to maximize the sensitivity. Each sample was analyzed in triplicate, using the thermic protocol described. Concerning the external reactions, mixtures contained 1× DreamTaq Green buffer (ThermoScientific, Life Technologies, Monza, Italy), 200 μM of each dNTP, 0.15 μM of each primer, 1U of DreamTaq Green DNA polymerase (ThermoScientific, Life Technologies, Monza, Italy), and 4 μL of DNA samples (approximately 250–500 ng of genomic DNA) in a final volume of 20 μL. The mixture of nested reactions contained 1× DreamTaq Green buffer (ThermoScientific, Life Technologies, Monza, Italy), 200 μM of each dNTP, 0.3 μM of each primer, 1U of DreamTaq Green DNA polymerase (ThermoScientific, Life Technologies, Monza, Italy), and 1 μL of PCR products, in a final volume of 20 μL. PCR products were run on 1.5% agarose gel containing 0.05% ethidium bromide in TBE buffer electrophoresis; bands were visualized under UV light on a transilluminator. Bands of expected size were excised from agarose gel, purified with a commercial kit (NucleoSpin^®^ Gel and PCR Clean-up kit, Macherey-Nagel GmbH and Co. KG, Düren, Germany), and sent for bidirectional sequencing to a commercial service (Eurofins MWG Operon, Ebersberg, Germany). Electropherograms were checked, and consensus sequences were manually assembled.

Sequences were compared to nucleotide sequences available in the GenBank using BLASTn (https://blast.ncbi.nlm.nih.gov/, accessed on 6 October 2020) and then aligned with sequences available in GenBank using the Mega6 software [23]. The sequences of the GRA6 locus were aligned with Type I—RH (JN649063.1), Type II—ME49(AF239285.1), and Type III—NED, CTG, C56 (AF239286.1, JX044207.1, DQ512729.1). The sequences of the BTUB locus were aligned with Type I—RH and GT1 (JX045508.1, JX045509.1), Type II—Beverly (AF249702.1), and Type III—CTG and C56 (JX045537.1, AF249703.1). The sequences of the altSAG2 locus were aligned with Type I—RH (JX045478.1), Type II—Beverly (AF249697.1), and Type III C56 and NED (AF249698.1, AF357579.1).

In addition, Nc5 real-time PCR positive amplicons were purified and sequenced, as described above. Obtained sequences, once cleaned up, were compared with the homologous nucleotide sequences available in GenBank databases, using the BLASTn for identity confirmation.

### 2.3. Statistical Analysis

The prevalence of *T. gondii* and *N. caninum* infection in the different species and taxonomic families of birds of prey included in the study and for the considered variables (age, WRC, reason for admission to the WRC, dietary habits, main migratory behavior) was calculated [24]. Chi-square test was used to verify the possible association between *T. gondii* infection and the following variables: taxonomic family; age (young, adult); WRC; reason for admission to the WRC (debilitation, trauma, other causes); dietary habits (mainly mammals, mainly birds, generalist species); and main migratory behavior (migratory, sedentary). The level of significance for independent variables was set to 0.05. Statistical analysis was performed by SPSS (version 19.0; SPSS, Chicago, IL, USA).

## 3. Results

*T. gondii* DNA was found in thirty-five (62.5%) samples examined by B1 real-time PCR. Positive control showed Ct and Tm values of 20.314 and 77.973, respectively; Ct and Tm values of positive samples ranged from 26.199 to 34.039, and from 77.795 to 78.480, respectively.

With regards to *N. caninum*, parasitic DNA was found in the brain of two birds (3.6%), out of the 56 examined. Positive control showed Ct and Tm values of 27.720 and 78.749, respectively. Positive samples showed Ct values of 32.736 and 34.180, and Tm values of 78.597 and 79.211. Sequencing of Nc5 real-time PCR amplicons produced two identical 76 bp sequences. BLASTn analysis confirmed *N. caninum* identity, showing that the obtained sequences had a 99–100% homology with *N. caninum* sequences deposited in GenBank (FJ464412, X84238).

The genotype determination was subsequently carried out by MLST analysis on the 35 samples that tested positive at the *T. gondii* B1 real-time PCR, by amplifying and sequencing regions of three target genes (GRA6, BTUB, and altSAG2). The amplification was possible only for part of the samples. In particular, for nine animals, it was not possible to obtain amplification for any target genes. Concerning the GRA6 gene, amplicons were obtained for eighteen raptors; high quality sequences were produced for only five samples. Further, only five samples were amplified at the BTUB target region, and four were successfully sequenced. The GRA6 and BTUB sequence alignment of the samples showed complete (100%) sequence homology with the clonal type II reference sequence included (Beverly for GRA6, and ME49 for BTUB) (Supplementary Tables S1 and S2). For both genes, any intraspecific nucleotide variation was not detected between the sequences examined (100% identity). Finally, concerning altSAG2 gene, 13 amplicons were obtained; all but one sample was successfully sequenced and the alignment of the sequences showed a homology with type II (ME49), but two single-nucleotide polymorphisms (SNPs) were identified. In all obtained sequences, double peaks (A/G) were detected at position 39 and 162 (Supplementary Table S3). The obtained sequence was submitted to the GenBank database under accession number MW590807.

The prevalence values of *T. gondii* and *N. caninum* were then calculated according to the considered individual and species-specific variables. Concerning *T. gondii*, the taxonomic family of Strigidae showed the highest prevalence of infection (68.8%), compared to Falconidae (63.2%) and Accipitridae (57.1%). Parasitic DNA was detected in both young (7/12, 58.3%) and adult animals (28/44, 63.6%). Positive raptors were hospitalized in both WRC1 and WRC2 (51.7% and 74.1%, respectively, out of the examined animals). The reasons for the admission to the WRC were then considered: *T. gondii* DNA was more frequently found in animals admitted for debilitation (4/5, 80%) than in those hospitalized for trauma (25/41, 61%) or for other causes (6/10, 60%). Finally, the dietary habits and the main migratory behavior were considered for each species included in the study. Raptor species that feed mainly on mammals were more frequently positive in *T. gondii* B1 real-time PCR (25/36, 69.4%) than those that feed mainly on birds (5/11, 45.4%) or compared to generalist species (5/9, 55.6%). Sedentary species showed higher prevalence of *T. gondii* infection than migratory species (25/37, 67.6%, and 19/19, 52.6%, respectively). Table 1 shows the biological characteristics considered (dietary habits and main migratory behavior) for each species included in the sampling, and the corresponding positivity values to B1 real-time PCR.

*N. caninum* infection was recorded in a young and in an adult common kestrel (*Falco tinnuculus*), both hospitalized for traumatic causes. The adult common kestrel (RAP56) was also co-infected by *T. gondii*.

Statistical analysis by Chi-Square test did not show any differences in the prevalence values of *T. gondii* infection among the considered categories (taxonomic family, age, WRC, reason for admission to the WRC, dietary habits, and main migratory behavior) (Table 2).

Since only two animals tested positive on molecular analysis, it was not possible to establish an association of *N. caninum* infection with the considered risk factors.

For each animal included in the study, individual data and results of molecular analysis are detailed in Supplementary Table S4.

## 4. Discussion

The results obtained in the present study demonstrated the presence of *T. gondii* and *N. caninum* in wild birds of prey admitted to WRCs, with the detection of the DNA of the two pathogens in 62.5% and 3.6% of the analyzed brain tissue samples, respectively. Due to the difficulties in carrying out this study in nature, WRC were chosen to carry out the sampling, as in other studies [6,7,8,25]. The possible bias related to the health status at the time of admission to the WRC was investigated and discussed below.

In the present study, a high prevalence of *T. gondii* infection was recorded, particularly in the species belonging to the Strigidae family (68.8%), followed by those belonging to the Falconidae (63.2%) and Accipitridae (57.1%) families. The prevalence was even higher considering the single host species, such as the Eurasian buzzard and the little owl (90% and 85.7%, respectively); possibly, ecology-related variables explain the inter-specific differences. However, the statistical analysis did not show significant associations between the prevalence data obtained and the considered variables, demonstrating the wide spread of the infection among the study population.

In the literature, several studies investigated the presence of this parasite in raptors, either through serological investigations or by using molecular techniques. Previous seroepidemiological studies conducted in Europe showed heterogeneous seroprevalence values: 29.8% in Spain, 36% in France, and 54% in Portugal of the raptors included in the sampling showed specific anti-*T. gondii* antibodies [7,8,25]. These differences might be due both to the lack of standardization of the serological techniques used in terms of tests and cut-off [4], but also due to the diverse host species included in the studies. Considering the Italian scenario, a recent seroepidemiological study conducted in the same study area of the present study found a *T. gondii* seroprevalence of 13.2% among wild birds of prey admitted to a WRC [6]. In addition, a similar seroprevalence value (26.1%) was recorded among the 238 kestrels captured-and-released at an airport site in the Emilia Romagna region (Northern Italy) [26]. A mismatch between the serological result reported by these two studies and the data obtained from the present molecular epidemiology study is therefore highlighted. In wild birds of prey, however, seroprevalence does not always correspond to positivity to the PCR on the brain [10], demonstrating that the association between seropositivity and the presence of *T. gondii* DNA in the tissues of these species is yet to be investigated. As evidence of the possible lifelong non-persistence of immunity, in the present study, no difference in the prevalence values was recorded between young and adult animals, as already reported in other wild species [27].

Considering molecular epidemiology, differences were reported among the available studies. Darwich et al. [9], by means of a nested PCR targeting the 529 bp repetitive fragment, found *T. gondii* DNA in the brain of only two positive raptors among the 136 examined in Spain. Nardoni et al. [10], using a nested PCR targeting the B1 gene, found *T. gondii* DNA in the brain and heart samples belonging to two positive *Falco tinnunculus*, among 15 raptors examined in Central Italy. A higher prevalence value was recorded by a recent study conducted in Turkey: *T. gondii* DNA was detected by Real Time PCR targeting the 529 bp repetitive fragment in the brain and heart samples of 92.3% out of 43 examined raptors [28]. These differences in prevalence values might be explained both by the sensitivity of the different molecular techniques, and the target genes use. In the present study, a Real Time PCR, generally having a higher sensitivity compared to end-point PCR, was used as screening and for targeting the B1 gene, which is present in multiple copies within the *T. gondii* genome, and is among the most widely used genes in molecular screening [22]. On the contrary, the PCR conducted in the genes used for genotyping, present in a single copy of the *T. gondii* genome, might be less sensitive. As in the present study, this often results in fewer genotyped samples than those tested positive in the screening PCR [28,29].

Furthermore, the molecular epidemiology studies conducted on avian species usually include various species of wild raptors, which, due to their different ethological behaviors, could be exposed to a different degree of risk of acquiring *T. gondii* infection. In fact, strict carnivorous or scavenger species are more at risk than generalist species [8]. Although there is a slightly higher prevalence of infection in species that normally feed on micro-mammals compared to species that prey on other birds or generalist species, since the difference is not statistically significant, the results obtained do not suggest that the dietary habits represents a risk factor for *T. gondii* infection for birds of prey. Generally, raptors as apex predators might be considered indicators of the spread of infection in the environment and in other animal species that share the same habitats and trophic chains.

Indeed, the presence of *T. gondii* in the study area was previously extensively reported with the detection of specific antibodies or parasitic DNA in different domestic [30,31,32] and wild host species [20,27], demonstrating the presence of both a domestic and a sylvatic cycle in this area. The animals admitted to the WCR are usually individuals living near urban centers; for this reason, they are more frequently subject to anthropogenic trauma (e.g., impact with windows and cars), and more easily identifiable and referable to WRCs.

All birds included in the study were admitted to WCRs, but then died or were euthanized as a result of various pathologies. Interestingly, even if this data were not statistically significant, a higher prevalence of *T. gondii* was detected in birds of prey admitted to a WCR due to their debilitation than in those hospitalized for other causes, suggesting an involvement of *T. gondii* in the deterioration of the general conditions of the animals up to the fatal outcome. Although few cases of clinical toxoplasmosis are reported in raptor species [11,12,13], this finding could suggest to veterinarians operating in WCRs to include toxoplasmosis in the differential diagnosis, especially in the case of debilitated birds of prey.

Given the high environmental contamination by *T. gondii* oocysts in densely populated areas [33], the finding of the infection in sedentary raptor species might be indicative of the prevalence of the parasite in the area where they live. On the contrary, long-range migratory species, could be used as indicators of *T. gondii* genetic variability on their migration route or in the country of destination, as they could acquire the infection during their migration. Consequently, an expected result could be a certain degree of difference between sedentary and migratory species, also with regard to the involved *T. gondii* genotypes. Karakavuk et al. [28] genotyped by microsatellite analysis a total of 14 strains, belonging to Type II (8 isolates), Type III (3 isolates), or atypical Types (3 isolates) in Turkey, a strategic area for the passage of migratory avian species. Indeed, a wide genetic variability is possible in the sylvatic cycle, especially in migratory species. On the contrary, all isolates successfully sequenced in the present study showed homology of sequence with Type II. On the other hand, Type II is the genotype most frequently found in Europe, both in domestic animals and in synanthropic or wild animals, including wild birds [34,35]. Interestingly, two SNPs were identified within the altSAG2 gene, which could represent an adaptation of the parasite to the raptor host species. However, further studies including more *T. gondii* isolates from raptors are needed to confirm this genetic trait of the parasite infecting raptors.

*N. caninum* in brain tissue of wild birds of prey was also investigated in this study. The presence of birds in farms as risk factors for neosporosis in cattle [36,37] suggests the involvement of avian species in the *N. caninum* cycle, even if the underlying biological mechanism and the epidemiological role of these species is still not fully elucidated.

Indeed, there are only few reports of natural *N. caninum* infections in domestic and wild birds, demonstrated both with the presence of specific antibodies and with the detection of the DNA of the parasite in the tissues of the hosts, as recently reviewed [5]. Considering in particular the birds of prey, attempts of experimental *N. caninum* infection have failed [38], while to the best of our knowledge the parasite DNA was previously demonstrated only in the brain tissue of a naturally infected buzzard [9]. In our study, *N. caninum* DNA was found in the brain of two common kestrels, one young and one adult animal, the second was also co-infected with *T. gondii*. These data, therefore, add a new species among the naturally intermediate hosts that can therefore acquire *N. caninum* infection. It is plausible that the two kestrels, preying mainly on micro-mammals, acquired the infection by ingesting infected small rodents. Micro-mammal species, such as house mice (*Mus musculus*) and field mice (*Apodemus sylvaticus*), are in fact among the suitable intermediate host species of both *T. gondii* and *N. caninum* [39,40], potentially representing the link between the sylvatic cycle, to which the birds of prey belong, and the domestic cycle involving dogs and cattle.

It is noteworthy to consider that in the study area, *N. caninum* is highly prevalent both in dairy cattle farms where it is recognized as an important cause of abortion [21], and in small ruminants [41]. Recent studies conducted on aborted bovine fetuses identified local *N. caninum* subpopulations in Northern Italian regions [42], and a significant association between the geographic distance of the farms and the genetic distance determined by multilocus-microsatellite genotyping was evidenced [21]. Therefore, multilocus microsatellite genotyping of *N. caninum* from other intermediate hosts, including wild birds, could be indicative of the spatial distribution and mutual connections between the parasite isolates from different species.

## 5. Conclusions

In conclusion, data obtained in the present study confirmed raptors as natural hosts of *T. gondii* and endorsed *F. tinnunculus* as a suitable intermediate host species for *N. caninum*, confirming the potential role of these animals in the sylvatic and potentially also in the domestic life cycle of these pathogens. Further studies of molecular epidemiology, as well as the isolation of pathogens, are necessary for understanding the actual role of birds of prey in the sylvatic cycle and, generally, in the epidemiology of *T. gondii* and *N. caninum*. Many species of birds of prey, such as kestrels, buzzards, nocturnal birds of prey, share large portions of their habitat with humans, and are showing an increasing trend towards urbanization in response to the expansion of the territory occupied by humans. Their position at the top of trophic chains, and their stable presence in urbanized areas, makes them ideal sentinel species for the circulation of *T. gondii*, a zoonotic parasite, and of *N. caninum*, impacting livestock production. The fundamental role of WRCs in the epidemiological surveillance of wildlife is therefore confirmed.

## Figures and Tables

**Table 1:** List of the species of raptors included in the sampling. The dietary habits and migratory behavior, and the corresponding positivity values to *Toxoplasma gondii* B1 real-time PCR is given for each species.

Family	Common Name (Species)	*T. gondii* B1 Real-Time PCR Positive/Examined (%)	Dietary Habits	Main Migratory Behavior
Accipitridae	northern goshawk (*Accipiter gentilis*)	0/1 (0%)	mainly birds	sedentary
	Eurasian sparrowhawk (*Accipiter nisus*)	3/7 (42.9%)	mainly birds	sedentary
	Eurasian buzzard (*Buteo buteo*)	9/10 (90%)	mainly mammals	sedentary
	black kite (*Milvus migrans*)	0/2 (0%)	generalist species	migratory
	European honey buzzard (*Pernis apivorus*)	0/1 (0%)	generalist species	migratory
		12/21 (57.1%)		
Falconidae	peregrine falcon (*Falco peregrinus*)	1/1 (100%)	mainly birds	sedentary
	Eurasian hobby (*Falco subbuteo*)	1/2 (50%)	mainly birds	migratory
	common kestrel (*Falco tinnunculus*)	10/16 (62.5%)	mainly mammals	migratory
		12/19 (63.2%)		
Strigidae	northern long-eared owl (*Asio otus*)	4/5 (80%)	mainly mammals	sedentary
	little owl (*Athene noctua*)	6/7 (85.7%)	generalist species	sedentary
	tawny owl (*Strix aluco*)	1/4 (25%)	mainly mammals	sedentary
		11/16 (68.7%)		

**Table 2:** Variables associated to *Toxoplasma gondii* infection in raptors from two Wildlife Recovery Centers (WRC) in Northern Italy.

Variable	Category	*T. gondii* B1 Real-Time PCR Positive/Examined (%)	*p*-Value ^a^
Taxonomic family	Accipitridae	12/21 (57.1%)	0.768
	Falconidae	12/19 (63.2%)	
	Strigidae	11/16 (68.8%)	
Age	young	7/12 (58.3%)	0.737
	adult	28/44 (63.6%)	
WRC	WRC1	15/29 (51.7%)	0.084
	WRC2	20/27 (74.1%)	
Reason for admission to the WRC	debilitation	4/5 (80%)	0.697
	trauma	25/41 (61%)	
	other causes	6/10 (60%)	
Dietary habits	mainly mammals	25/36 (69.4%)	0.318
	mainly bird	5/11 (45.5%)	
	generalist species	5/9 (55.6%)	
Migratory behavior	migratory	10/19 (52.6%)	0.274
	sedentary	25/37 (67.6%)	

^a^ Pearson’s Chi-Square test.

## Data Availability

Data supporting the conclusions of this article are included within the article and its Supplementary Materials.

## Supplementary Materials

The following are available online at https://www.mdpi.com/article/10.3390/microorganisms9040736/s1. Table S1: Alignment of GRA6, Table S2: Alignment of BTUB, Table S3: Alignment of altSAG2, and Table S4: Individual data and results of molecular analysis.
