# Supplementary material for: Molecular Survey on Toxoplasma gondii and Neospora caninum Infection in Wild Birds of Prey Admitted to Recovery Centers in Northern Italy
Source: Microorganisms. 2021 Apr 1;9(4):736. doi: 10.3390/microorganisms9040736 (PMC8065797; doi:10.3390/microorganisms9040736)
Supplement: Supplementary file 1 [file microorganisms-09-00736-s001.zip › Additional file 1 - allineamento gra6 rapaci.docx]

Table S1:

Alignment of GRA6

10 20 30 40 50

....|....| ....|....| ....|....| ....|....| ....|....|

JN649063 Type I - RH TTTCCGAGCA GGTGACCTGG GTCGCTTTTT TGAAACAGCA GGAAAACAGC

AF239285 Type II - ME49 .......... .......... .......... .......... ..........

DQ512729 Type III - C56 .......... .......... .......... .......... ..........

JX044207 Type III - CTG .......... .......... .......... .......... ..........

AF239286 Type III - NED .......... .......... .......... .......... ..........

RAP08 .......... .......... .......... .......... ..........

RAP33 .......... .......... .......... .......... ..........

RAP36 .......... .......... .......... .......... ..........

RAP49 .......... .......... .......... .......... ..........

RAP56 .......... .......... .......... .......... ..........

60 70 80 90 100

....|....| ....|....| ....|....| ....|....| ....|....|

JN649063 Type I - RH TTCGTGGTGC CACGTAGCGT GCTTGTTGGC GACTACCTTT TTTTCTTGGG

AF239285 Type II - ME49 .......... .......... .......... .......... ..........

DQ512729 Type III - C56 .......... .......... .......... .......... ..........

JX044207 Type III - CTG .......... .......... .......... .......... ..........

AF239286 Type III - NED .......... .......... .......... .......... ..........

RAP08 .......... .......... .......... .......... ..........

RAP33 .......... .......... .......... .......... ..........

RAP36 .......... .......... .......... .......... ..........

RAP49 .......... .......... .......... .......... ..........

RAP56 .......... .......... .......... .......... ..........

110 120 130 140 150

....|....| ....|....| ....|....| ....|....| ....|....|

JN649063 Type I - RH AGTGTCGGCG AAATGGCACA CGGTGGCATC CATCTGAGGC AGAAGCGTAA

AF239285 Type II - ME49 .......... .......... .......... T......... ..........

DQ512729 Type III - C56 .......... .......... .......... .......... ..........

JX044207 Type III - CTG .......... .......... .......... .......... ..........

AF239286 Type III - NED .......... .......... .......... .......... ..........

RAP08 .......... .......... .......... T......... ..........

RAP33 .......... .......... .......... T......... ..........

RAP36 .......... .......... .......... T......... ..........

RAP49 .......... .......... .......... T......... ..........

RAP56 .......... .......... .......... T......... ..........

160 170 180 190 200

....|....| ....|....| ....|....| ....|....| ....|....|

JN649063 Type I - RH CTTCTGTCCT GTAACTGTCT CCACAGTTGC TGTGGTCTTT GTAGTCTTCA

AF239285 Type II - ME49 .......... T......... .......... .......... ..........

DQ512729 Type III - C56 .......... T......... .......... .......... .....T....

JX044207 Type III - CTG .......... T......... .......... .......... .....T....

AF239286 Type III - NED .......... T......... .......... .......... .....T....

RAP08 .......... T......... .......... .......... ..........

RAP33 .......... T......... .......... .......... ..........

RAP36 .......... T......... .......... .......... ..........

RAP49 .......... T......... .......... .......... ..........

RAP56 .......... T......... .......... .......... ..........

210 220 230 240 250

....|....| ....|....| ....|....| ....|....| ....|....|

JN649063 Type I - RH TGGGTGTACT CGTCAATTCG TTGGGTGGAG TCGCTGTCGC AGCAGACAGC

AF239285 Type II - ME49 .......... .......... .......... .......... ..........

DQ512729 Type III - C56 .......... .......... .......... .......... ..........

JX044207 Type III - CTG .......... .......... .......... .......... ..........

AF239286 Type III - NED .......... .......... .......... .......... ..........

RAP08 .......... .......... .......... .......... ..........

RAP33 .......... .......... .......... .......... ..........

RAP36 .......... .......... .......... .......... ..........

RAP49 .......... .......... .......... .......... ..........

RAP56 .......... .......... .......... .......... ..........

260 270 280 290 300

....|....| ....|....| ....|....| ....|....| ....|....|

JN649063 Type I - RH GGTGGTGTTA AGCAGACCCC TTCGGAAACC GGTTCGAGCG GTGGACAGCA

AF239285 Type II - ME49 .......... G......... .......... .......... ..........

DQ512729 Type III - C56 .A........ .......... .......... .......... ..........

JX044207 Type III - CTG .A........ .......... .......... .......... ..........

AF239286 Type III - NED .A........ .......... .......... .......... ..........

RAP08 .......... G......... .......... .......... ..........

RAP33 .......... G......... .......... .......... ..........

RAP36 .......... G......... .......... .......... ..........

RAP49 .......... G......... .......... .......... ..........

RAP56 .......... G......... .......... .......... ..........

310 320 330 340

....|....| ....|....| ....|....| ....|....| ....

JN649063 Type I - RH AGAAGCAGTG GGGACCACTG AAGACTATGT CAACTCTTCG GCGA

AF239285 Type II - ME49 .......... .......... .......... .......... ....

DQ512729 Type III - C56 .......... .......... .......... .......... ....

JX044207 Type III - CTG .......... .......... .......... .......... ....

AF239286 Type III - NED .......... .......... .......... .......... ....

RAP08 .......... .......... .......... .......... ....

RAP33 .......... .......... .......... .......... ....

RAP36 .......... .......... .......... .......... ....

RAP49 .......... .......... .......... .......... ....

RAP56 .......... .......... .......... .......... ....
