# Supplementary material for: Molecular Survey on Toxoplasma gondii and Neospora caninum Infection in Wild Birds of Prey Admitted to Recovery Centers in Northern Italy
Source: Microorganisms. 2021 Apr 1;9(4):736. doi: 10.3390/microorganisms9040736 (PMC8065797; doi:10.3390/microorganisms9040736)
Supplement: Supplementary file 1 [file microorganisms-09-00736-s001.zip › Additional file 2 - allineamento btub rapaci.docx]

Table S2:

Alignment of BTUB

10 20 30 40 50

....|....| ....|....| ....|....| ....|....| ....|....|

JX045508 Type I - RH .......... .......... .......... .......... ..........

JX045509 Type I - GT1 GAGGTCATCT CGGACGAACA CGGCATTGAT CCGGTGAGGG AGAACGGGTT

AF249702 Type II - BEVERLEY .......... .......... .......... .......... ..........

JX045537 Type III - CTG .......... .......... .......... .......... ..........

AF249703 Type III - C56 .......... .......... .......... .......... ..........

RAP08 .......... .......... .......... .......... ..........

RAP33 .......... .......... .......... .......... ..........

RAP36 .......... .......... .......... .......... ..........

RAP49 .......... .......... .......... .......... ..........

60 70 80 90 100

....|....| ....|....| ....|....| ....|....| ....|....|

JX045508 Type I - RH .......... .......... .......... .......... ..........

JX045509 Type I - GT1 CCGGAGTGCG GGTTCCCGGA CGCTGTCTTT TGTCCTGTAT TCTGCGTGAC

AF249702 Type II - BEVERLEY .......... .......... .......... .......... ..........

JX045537 Type III - CTG .......... .......... .......... .......... ..........

AF249703 Type III - C56 .......... .......... .......... .......... ..........

RAP08 .......... .......... .......... .......... ..........

RAP33 .......... .......... .......... .......... ..........

RAP36 .......... .......... .......... .......... ..........

RAP49 .......... .......... .......... .......... ..........

110 120 130 140 150

....|....| ....|....| ....|....| ....|....| ....|....|

JX045508 Type I - RH .......... .......... .......... .......... ..........

JX045509 Type I - GT1 AGCTTCGCCA GTGTAAATCG AGCCGTTTTC CCTGTGGAGA ATCGCGGAGA

AF249702 Type II - BEVERLEY .......... ........G. .......... .......... ..........

JX045537 Type III - CTG .......... .......... .......... .......... ..........

AF249703 Type III - C56 .......... .......... .......... .......... ..........

RAP08 .......... ........G. .......... .......... ..........

RAP33 .......... ........G. .......... .......... ..........

RAP36 .......... ........G. .......... .......... ..........

RAP49 .......... ........G. .......... .......... ..........

160 170 180 190 200

....|....| ....|....| ....|....| ....|....| ....|....|

JX045508 Type I - RH .......... .......... .......... .......... ..........

JX045509 Type I - GT1 ATGGACGAGT TTTCCGAGCT CGCAGGTGCC ACCCTCCACC TCGACGCAAC

AF249702 Type II - BEVERLEY .....G..C. .......... .......... .......... ..........

JX045537 Type III - CTG .......... .......... .......... .......... ..........

AF249703 Type III - C56 .......... .......... .......... .......... ..........

RAP08 .....G..C. .......... .......... .......... ..........

RAP33 .....G..C. .......... .......... .......... ..........

RAP36 .....G..C. .......... .......... .......... ..........

RAP49 .....G..C. .......... .......... .......... ..........

210 220 230 240 250

....|....| ....|....| ....|....| ....|....| ....|....|

JX045508 Type I - RH .......... .......... .......... .......... ..........

JX045509 Type I - GT1 CAAGTGCACG CACATTTGCC GGGTGGTGAC CCTAAGACAC CGCAGGTCTA

AF249702 Type II - BEVERLEY .......... .......... .......... .......... ..........

JX045537 Type III - CTG .......... .......... .......... .......... ..........

AF249703 Type III - C56 .......... .......... .......... .......... ..........

RAP08 .......... .......... .......... .......... ..........

RAP33 .......... .......... .......... .......... ..........

RAP36 .......... .......... .......... .......... ..........

RAP49 .......... .......... .......... .......... ..........

260 270 280 290 300

....|....| ....|....| ....|....| ....|....| ....|....|

JX045508 Type I - RH .......... .......... .......... .......... ..........

JX045509 Type I - GT1 CCTGCGCGTT TTTTTCAGTC TTTGCACACA GTTGCACCGA AAGTCATGTT

AF249702 Type II - BEVERLEY .......... .......... .......... .......... ..........

JX045537 Type III - CTG .......... .......... .......... .......... ..........

AF249703 Type III - C56 .......... .......... .......... .......... ..........

RAP08 .......... .......... .......... .......... ..........

RAP33 .......... .......... .......... .......... ..........

RAP36 .......... .......... .......... .......... ..........

RAP49 .......... .......... .......... .......... ..........

310 320 330 340 350

....|....| ....|....| ....|....| ....|....| ....|....|

JX045508 Type I - RH .......... .......... .......... .......... ..........

JX045509 Type I - GT1 TTTTGCGAAG ACATCGGTTG TTCTGGTGGG GGAATACTTC ACTCCTGTTC

AF249702 Type II - BEVERLEY .......... ........C. .......... .......... ..........

JX045537 Type III - CTG .......... ........C. .......... .......... ..........

AF249703 Type III - C56 .......... ........C. .......... .......... ..........

RAP08 .......... ........C. .......... .......... ..........

RAP33 .......... ........C. .......... .......... ..........

RAP36 .......... ........C. .......... .......... ..........

RAP49 .......... ........C. .......... .......... ..........

360 370 380 390 400

....|....| ....|....| ....|....| ....|....| ....|....|

JX045508 Type I - RH .......... .......... .......... .......... ..........

JX045509 Type I - GT1 GCGCCTATGT GCGCAGACAG GTGTCCACCC TCCGCATACG GGCGTCCGGG

AF249702 Type II - BEVERLEY .......... .......... .......... .......... ..........

JX045537 Type III - CTG .......... .......... .......... .......... ..........

AF249703 Type III - C56 .......... .......... .......... .......... ..........

RAP08 .......... .......... .......... .......... ..........

RAP33 .......... .......... .......... .......... ..........

RAP36 .......... .......... .......... .......... ..........

RAP49 .......... .......... .......... .......... ..........

410

....|....| .

JX045508 Type I - RH .......... .

JX045509 Type I - GT1 TGTTCCTACA A

AF249702 Type II - BEVERLEY .......... .

JX045537 Type III - CTG .......... .

AF249703 Type III - C56 .......... .

RAP08 .......... .

RAP33 .......... .

RAP36 .......... .

RAP49 .......... .
