# Supplementary material for: Molecular Survey on Toxoplasma gondii and Neospora caninum Infection in Wild Birds of Prey Admitted to Recovery Centers in Northern Italy
Source: Microorganisms. 2021 Apr 1;9(4):736. doi: 10.3390/microorganisms9040736 (PMC8065797; doi:10.3390/microorganisms9040736)
Supplement: Supplementary file 1 [file microorganisms-09-00736-s001.zip › Additional file 3 - allineamento altSAG2 rapaci.docx]

Table S3:

Alignment of altSAG2

10 20 30 40 50

....|....| ....|....| ....|....| ....|....| ....|....|

JX045478 Type I - RH ---------- ---------- ---------- ------ATGA GTTTCTCAAA

AF249697 Type II - BEVERLEY CTTCTGTCTC GTTCCAATCT TTGTCTTGTC GGAACT.... ..........

AF357579 Type III - NED CTTCTGTCTC GTTCCAATCT TTGTCTTGTC GGAACT.... ..........

AF249698 Type III - C56 CTTCTGTCTC GTTCCAATCT TTGTCTTGTC GGAACT.... ..........

RAP12 CTTCTGTCTC GTTCCAATCT TTGTCTTGTC GGAACT..R. ..........

RAP17 CTTCTGTCTC GTTCCAATCT TTGTCTTGTC GGAACT..R. ..........

RAP18 CTTCTGTCTC GTTCCAATCT TTGTCTTGTC GGAACT..R. ..........

RAP24 CTTCTGTCTC GTTCCAATCT TTGTCTTGTC GGAACT..R. ..........

RAP27 CTTCTGTCTC GTTCCAATCT TTGTCTTGTC GGAACT..R. ..........

RAP28 CTTCTGTCTC GTTCCAATCT TTGTCTTGTC GGAACT..R. ..........

RAP33 CTTCTGTCTC GTTCCAATCT TTGTCTTGTC GGAACT..R. ..........

RAP36 CTTCTGTCTC GTTCCAATCT TTGTCTTGTC GGAACT..R. ..........

RAP42 CTTCTGTCTC GTTCCAATCT TTGTCTTGTC GGAACT..R. ..........

RAP49 CTTCTGTCTC GTTCCAATCT TTGTCTTGTC GGAACT..R. ..........

RAP53 CTTCTGTCTC GTTCCAATCT TTGTCTTGTC GGAACT..R. ..........

RAP55 CTTCTGTCTC GTTCCAATCT TTGTCTTGTC GGAACT..R. ..........

60 70 80 90 100

....|....| ....|....| ....|....| ....|....| ....|....|

JX045478 Type I - RH GACCACGAGC CTAGCGTCGC TAGCGCTCAC GGGCTTGTTT GTTGTGTTCA

AF249697 Type II - BEVERLEY .......... .......... .......... .......... ..........

AF357579 Type III - NED .......... .......... .......... .......... ..........

AF249698 Type III - C56 .......... .......... .......... .......... ..........

RAP12 .......... .......... .......... .......... ..........

RAP17 .......... .......... .......... .......... ..........

RAP18 .......... .......... .......... .......... ..........

RAP24 .......... .......... .......... .......... ..........

RAP27 .......... .......... .......... .......... ..........

RAP28 .......... .......... .......... .......... ..........

RAP33 .......... .......... .......... .......... ..........

RAP36 .......... .......... .......... .......... ..........

RAP42 .......... .......... .......... .......... ..........

RAP49 .......... .......... .......... .......... ..........

RAP53 .......... .......... .......... .......... ..........

RAP55 .......... .......... .......... .......... ..........

110 120 130 140 150

....|....| ....|....| ....|....| ....|....| ....|....|

JX045478 Type I - RH AGTTCGCTCT TGCGTCCACC ACCGAGACGC CAGCGCCCAT TGAGTGCACT

AF249697 Type II - BEVERLEY .......... .......... .......... .......... ..........

AF357579 Type III - NED .......... .......... .......... .......... ..........

AF249698 Type III - C56 .......... .......... .......... .......... ..........

RAP12 .......... .......... .......... .......... ..........

RAP17 .......... .......... .......... .......... ..........

RAP18 .......... .......... .......... .......... ..........

RAP24 .......... .......... .......... .......... ..........

RAP27 .......... .......... .......... .......... ..........

RAP28 .......... .......... .......... .......... ..........

RAP33 .......... .......... .......... .......... ..........

RAP36 .......... .......... .......... .......... ..........

RAP42 .......... .......... .......... .......... ..........

RAP49 .......... .......... .......... .......... ..........

RAP53 .......... .......... .......... .......... ..........

RAP55 .......... .......... .......... .......... ..........

160 170 180 190 200

....|....| ....|....| ....|....| ....|....| ....|....|

JX045478 Type I - RH GCCGGCGCAA CGAAGACTGT TGATGCACCC TCCAGTGGTT CCGTTGTCTT

AF249697 Type II - BEVERLEY .......... .......... ...G...... .......... ..........

AF357579 Type III - NED .......... .......... .......... .......... ..........

AF249698 Type III - C56 .......... .......... .......... .......... ..........

RAP12 .......... .R........ ...G...... .......... ..........

RAP17 .......... .R........ ...G...... .......... ..........

RAP18 .......... .R........ ...G...... .......... ..........

RAP24 .......... .R........ ...G...... .......... ..........

RAP27 .......... .R........ ...G...... .......... ..........

RAP28 .......... .R........ ...G...... .......... ..........

RAP33 .......... .R........ ...G...... .......... ..........

RAP36 .......... .R........ ...G...... .......... ..........

RAP42 .......... .R........ ...G...... .......... ..........

RAP49 .......... .R........ ...G...... .......... ..........

RAP53 .......... .R........ ...G...... .......... ..........

RAP55 .......... .R........ ...G...... .......... ..........

210 220 230 240 250

....|....| ....|....| ....|....| ....|....| ....|....|

JX045478 Type I - RH CCAATGTGGG GATAAACTAA CCATCAGTCC CAGTGGCGAA GGTGATGTCT

AF249697 Type II - BEVERLEY .......... .......... .......... .......... ..........

AF357579 Type III - NED .......... .......... .......... .......... ..........

AF249698 Type III - C56 .......... .......... .......... .......... ..........

RAP12 .......... .......... .......... .......... ..........

RAP17 .......... .......... .......... .......... ..........

RAP18 .......... .......... .......... .......... ..........

RAP24 .......... .......... .......... .......... ..........

RAP27 .......... .......... .......... .......... ..........

RAP28 .......... .......... .......... .......... ..........

RAP33 .......... .......... .......... .......... ..........

RAP36 .......... .......... .......... .......... ..........

RAP42 .......... .......... .......... .......... ..........

RAP49 .......... .......... .......... .......... ..........

RAP53 .......... .......... .......... .......... ..........

RAP55 .......... .......... .......... .......... ..........

260 270 280 290 300

....|....| ....|....| ....|....| ....|....| ....|....|

JX045478 Type I - RH TTTATGGCAA GGAATGCACA GACTCGAGGA AGTTGACGAC TGTCCTTCCA

AF249697 Type II - BEVERLEY .......... .......... .......... .......... ..........

AF357579 Type III - NED .......... .......... .......... .......... ..........

AF249698 Type III - C56 .......... .......... .......... .......... ..........

RAP12 .......... .......... .......... .......... ..........

RAP17 .......... .......... .......... .......... ..........

RAP18 .......... .......... .......... .......... ..........

RAP24 .......... .......... .......... .......... ..........

RAP27 .......... .......... .......... .......... ..........

RAP28 .......... .......... .......... .......... ..........

RAP33 .......... .......... .......... .......... ..........

RAP36 .......... .......... .......... .......... ..........

RAP42 .......... .......... .......... .......... ..........

RAP49 .......... .......... .......... .......... ..........

RAP53 .......... .......... .......... .......... ..........

RAP55 .......... .......... .......... .......... ..........

310 320 330 340 350

....|....| ....|....| ....|....| ....|....| ....|....|

JX045478 Type I - RH GGTGCGGTCT TGACAGCTAA GGTCCAGCAG CCCGCGAAAG GTCCTGCTAC

AF249697 Type II - BEVERLEY .......... ...A...... ....G..... ...C...... ..........

AF357579 Type III - NED .......... .......... .......... .......... ..........

AF249698 Type III - C56 .......... .......... .......... .......... ..........

RAP12 .......... ...A...... ....G..... ...C...... ..........

RAP17 .......... ...A...... ....G..... ...C...... ..........

RAP18 .......... ...A...... ....G..... ...C...... ..........

RAP24 .......... ...A...... ....G..... ...C...... ..........

RAP27 .......... ...A...... ....G..... ...C...... ..........

RAP28 .......... ...A...... ....G..... ...C...... ..........

RAP33 .......... ...A...... ....G..... ...C...... ..........

RAP36 .......... ...A...... ....G..... ...C...... ..........

RAP42 .......... ...A...... ....G..... ...C...... ..........

RAP49 .......... ...A...... ....G..... ...C...... ..........

RAP53 .......... ...A...... ....G..... ...C...... ..........

RAP55 .......... ...A...... ....G..... ...C...... ..........

360 370 380 390 400

....|....| ....|....| ....|....| ....|....| ....|....|

JX045478 Type I - RH CTACACACTG TCTTACGACG GTACTCCCGA GAAACCTCAG GTTCTCTGTT

AF249697 Type II - BEVERLEY .......... .......... .......... .......... ..........

AF357579 Type III - NED .......... .......... .......... .......... ..........

AF249698 Type III - C56 .......... .......... .......... .......... ..........

RAP12 .......... .......... .......... .......... ..........

RAP17 .......... .......... .......... .......... ..........

RAP18 .......... .......... .......... .......... ..........

RAP24 .......... .......... .......... .......... ..........

RAP27 .......... .......... .......... .......... ..........

RAP28 .......... .......... .......... .......... ..........

RAP33 .......... .......... .......... .......... ..........

RAP36 .......... .......... .......... .......... ..........

RAP42 .......... .......... .......... .......... ..........

RAP49 .......... .......... .......... .......... ..........

RAP53 .......... .......... .......... .......... ..........

RAP55 .......... .......... .......... .......... ..........

410 420 430 440 450

....|....| ....|....| ....|....| ...|....| ....|....|.

JX045478 Type I - RH ACAAGTGCGT TGCCGAAGCA GGTGCTCCCG CTGGTCGAA ATAATGATGG

AF249697 Type II - BEVERLEY .......... .......... .......... ......... ..........

AF357579 Type III - NED .......... .......... .......... ......... ..........

AF249698 Type III - C56 .......... .......... .......... ......... ..........

RAP12 .......... .......... .......... ......... ..........

RAP17 .......... .......... .......... ......... ..........

RAP18 .......... .......... .......... ......... ..........

RAP24 .......... .......... .......... ......... ..........

RAP27 .......... .......... .......... ......... ..........

RAP28 .......... .......... .......... ......... ..........

RAP33 .......... .......... .......... ......... ..........

RAP36 .......... .......... .......... ......... ..........

RAP42 .......... .......... .......... ......... ..........

RAP49 .......... .......... .......... ......... ..........

RAP53 .......... .......... .......... ......... ..........

RAP55 .......... .......... .......... ......... ..........

460 470 480 490 500

...|....| ....|....| ....|....| ....|....| ....|....|.

JX045478 Type I - RH ---TTCTAGC GCTCCGACGC CTAAAGACTG CAAACTCATT GTTCGCGTTC

AF249697 Type II - BEVERLEY TGG....... .......... .......... .......... ..........

AF357579 Type III - NED ---....... .......... .......... .......... ..........

AF249698 Type III - C56 ---....... .......... .......... .......... ..........

RAP12 TGG....... .......... .......... .......... ..........

RAP17 TGG....... .......... .......... .......... ..........

RAP18 TGG....... .......... .......... .......... ..........

RAP24 TGG....... .......... .......... .......... ..........

RAP27 TGG....... .......... .......... .......... ..........

RAP28 TGG....... .......... .......... .......... ..........

RAP33 TGG....... .......... .......... .......... ..........

RAP36 TGG....... .......... .......... .......... ..........

RAP42 TGG....... .......... .......... .......... ..........

RAP49 TGG....... .......... .......... .......... ..........

RAP53 TGG....... .......... .......... .......... ..........

RAP55 TGG....... .......... .......... .......... ..........

510

...|....| ....|..

JX045478 Type I - RH CGGGTGCCGA TGGCCG

AF249697 Type II - BEVERLEY .......... ......

AF357579 Type III - NED .......... ......

AF249698 Type III - C56 .......... ......

RAP12 .......... ......

RAP17 .......... ......

RAP18 .......... ......

RAP24 .......... ......

RAP27 .......... ......

RAP28 .......... ......

RAP33 .......... ......

RAP36 .......... ......

RAP42 .......... ......

RAP49 .......... ......

RAP53 .......... ......

RAP55 .......... ......
