# Supplementary material for: Molecular Survey on Toxoplasma gondii and Neospora caninum Infection in Wild Birds of Prey Admitted to Recovery Centers in Northern Italy
Source: Microorganisms. 2021 Apr 1;9(4):736. doi: 10.3390/microorganisms9040736 (PMC8065797; doi:10.3390/microorganisms9040736)
Supplement: Supplementary file 1 [file microorganisms-09-00736-s001.zip › microorganisms-1126011-supplementary.docx]

**Supplementary table S4*.* Individual data and results of *Toxoplasma gondii* B1 real-time PCR, *T. gondii* genotyping (MLST) of selected genes, and *Neospora caninum* Nc5 real-time PCR for each raptor included in the study.**

| **ID** | **Species** | **family** | **age** | **WRC** | **reason for admission to the WRC** | **dietary habits** | **Main migratory behavior** | **B1 real-time PCR CT values ^*^** | **GENOTYPING ^*^** | | | **Nc5 real-time PCR CT values ^*^** |
| --- | --- | --- | --- | --- | --- | --- | --- | --- | --- | --- | --- | --- |
|  |  |  |  |  |  |  |  |  | **GRA6** | **BTUB** | **altSAG2** |  |
| RAP30 | *Accipiter gentilis* | Accipitridae | adult | WRC2 | trauma | mainly birds | Sedentary | N |  |  |  | N |
| RAP3 | *Accipiter nisus* | Accipitridae | adult | WRC1 | other causes | mainly birds | Sedentary | 28.828 | N | N | N | N |
| RAP9 | *Accipiter nisus* | Accipitridae | young | WRC1 | trauma | mainly birds | Sedentary | N |  |  |  | N |
| RAP11 | *Accipiter nisus* | Accipitridae | adult | WRC1 | trauma | mainly birds | Sedentary | 28.484 | N | N | N | N |
| RAP13 | *Accipiter nisus* | Accipitridae | adult | WRC1 | trauma | mainly birds | Sedentary | N |  |  |  | N |
| RAP15 | *Accipiter nisus* | Accipitridae | adult | WRC1 | trauma | mainly birds | Sedentary | 29.645 | N | N | N | N |
| RAP37 | *Accipiter nisus* | Accipitridae | adult | WRC2 | trauma | mainly birds | Sedentary | N |  |  |  | N |
| RAP48 | *Accipiter nisus* | Accipitridae | adult | WRC1 | trauma | mainly birds | Sedentary | N |  |  |  | N |
| RAP14 | *Buteo buteo* | Accipitridae | young | WRC1 | trauma | mainly mammals | Sedentary | 26.199 | n.d. | N | N | N |
| RAP16 | *Buteo buteo* | Accipitridae | adult | WRC2 | trauma | mainly mammals | Sedentary | 28.471 | N | N | N | N |
| RAP21 | *Buteo buteo* | Accipitridae | adult | WRC1 | trauma | mainly mammals | Sedentary | 28.744 | n.d. | N | N | N |
| RAP23 | *Buteo buteo* | Accipitridae | adult | WRC1 | other causes | mainly mammals | Sedentary | N |  |  |  | N |
| RAP27 | *Buteo buteo* | Accipitridae | adult | WRC1 | trauma | mainly mammals | Sedentary | 20.143 | N | N | Type II | N |
| RAP34 | *Buteo buteo* | Accipitridae | adult | WRC2 | other causes | mainly mammals | Sedentary | 27.828 | N | N | N | N |
| RAP36 | *Buteo buteo* | Accipitridae | adult | WRC2 | debilitation | mainly mammals | Sedentary |  | Type II | Type II | Type II | N |
| RAP40 | *Buteo buteo* | Accipitridae | adult | WRC2 | debilitation | mainly mammals | Sedentary | 28.893 | n.d. | N | N | N |
| RAP41 | *Buteo buteo* | Accipitridae | adult | WRC2 | debilitation | mainly mammals | Sedentary | 30.740 | n.d. | N | N | N |
| RAP45 | *Buteo buteo* | Accipitridae | adult | WRC1 | trauma | mainly mammals | Sedentary | 27.319 | n.d. | N | N | N |
| RAP4 | *Milvus migrans* | Accipitridae | adult | WRC1 | trauma | generalist species | Migratory | N |  |  |  | N |
| RAP5 | *Milvus migrans* | Accipitridae | adult | WRC1 | trauma | generalist species | Migratory | N |  |  |  | N |
| RAP10 | *Pernis apivorus* | Accipitridae | young | WRC1 | trauma | generalist species | migratory | N |  |  |  | N |
| RAP38 | *Falco peregrinus* | Falconidae | adult | WRC2 | trauma | mainly birds | Sedentary | 29.350 | n.d. | N | N | N |
| RAP19 | *Falco subbuteo* | Falconidae | adult | WRC1 | trauma | mainly birds | Migratory | N |  |  |  | N |
| RAP53 | *Falco subbuteo* | Falconidae | adult | WRC2 | trauma | mainly birds | Migratory | 30.163 | N | N | Type II | N |
| RAP1 | *Falco tinnunculus* | Falconidae | adult | WRC1 | trauma | mainly mammals | Migratory | 33.094 | N | N | n.d. | N |
| RAP7 | *Falco tinnunculus* | Falconidae | young | WRC1 | other causes | mainly mammals | Sedentary | N |  |  |  | N |
| RAP8 | *Falco tinnunculus* | Falconidae | young | WRC1 | other causes | mainly mammals | Sedentary | N | Type II | Type II | N | N |
| RAP20 | *Falco tinnunculus* | Falconidae | adult | WRC2 | trauma | mainly mammals | Migratory | 30.300 | N | N | N | N |
| RAP24 | *Falco tinnunculus* | Falconidae | young | WRC1 | trauma | mainly mammals | Migratory | 26.364 | N | N | Type II | N |
| RAP26 | *Falco tinnunculus* | Falconidae | young | WRC1 | trauma | mainly mammals | Migratory | N |  |  |  | 32.736 |
| RAP31 | *Falco tinnunculus* | Falconidae | adult | WRC2 | trauma | mainly mammals | Migratory | N |  |  |  | N |
| RAP39 | *Falco tinnunculus* | Falconidae | adult | WRC2 | other causes | mainly mammals | Migratory | N |  |  |  | N |
| RAP43 | *Falco tinnunculus* | Falconidae | adult | WRC2 | trauma | mainly mammals | Migratory | 28.324 | n.d. | N | N | N |
| RAP44 | *Falco tinnunculus* | Falconidae | young | WRC1 | trauma | mainly mammals | Migratory | 29.874 | n.d. | N | N | N |
| RAP46 | *Falco tinnunculus* | Falconidae | adult | WRC1 | other causes | mainly mammals | Migratory | N |  |  |  | N |
| RAP47 | *Falco tinnunculus* | Falconidae | adult | WRC1 | trauma | mainly mammals | Migratory | 28.428 | n.d. | N | N | N |
| RAP52 | *Falco tinnunculus* | Falconidae | adult | WRC2 | trauma | mainly mammals | Migratory | 31.595 | n.d. | N | N | N |
| RAP54 | *Falco tinnunculus* | Falconidae | adult | WRC2 | trauma | mainly mammals | migratory | N |  |  |  | N |
| RAP55 | *Falco tinnunculus* | Falconidae | adult | WRC2 | trauma | mainly mammals | Migratory | 29.614 | n.d. | N | Type II | N |
| RAP56 | *Falco tinnunculus* | Falconidae | adult | WRC2 | trauma | mainly mammals | Migratory | 32.341 | Type II | N | N | 34.180 |
| RAP2 | *Asio otus* | Strigidae | adult | WRC1 | trauma | mainly mammals | Sedentary | 34.039 | N | N | N. | N |
| RAP6 | *Asio otus* | Strigidae | adult | WRC1 | trauma | mainly mammals | Sedentary | N |  |  |  | N |
| RAP17 | *Asio otus* | Strigidae | young | WRC1 | other causes | mainly mammals | sedentary | 28.185 | n.d. | N | Type II | N |
| RAP18 | *Asio otus* | Strigidae | adult | WRC2 | trauma | mainly mammals | Sedentary | 27.458 | n.d. | N | Type II | N |
| RAP51 | *Asio otus* | Strigidae | young | WRC2 | trauma | mainly mammals | Sedentary | 31.488 | N | n.d. | N | N |
| RAP12 | *Athene noctua* | Strigidae | young | WRC1 | trauma | generalist species | Sedentary | 29.018 | N | N | Type II | N |
| RAP28 | *Athene noctua* | Strigidae | adult | WRC2 | trauma | mainly mammals | Sedentary | 27.782 | N | N | Type II | N |
| RAP32 | *Athene noctua* | Strigidae | adult | WRC2 | debilitation | generalist species | Sedentary | N |  |  |  | N |
| RAP33 | *Athene noctua* | Strigidae | adult | WRC2 | debilitation | generalist species | Sedentary |  | Type II | Type II | Type II | N |
| RAP35 | *Athene noctua* | Strigidae | adult | WRC2 | other causes | generalist species | Sedentary | 28.787 | n.d. | N | N | N |
| RAP49 | *Athene noctua* | Strigidae | adult | WRC2 | trauma | generalist species | Sedentary |  | Type II | Type II | Type II | N |
| RAP50 | *Athene noctua* | Strigidae | adult | WRC2 | trauma | generalist species | Sedentary | 32.904 |  |  |  | N |
| RAP22 | *Strix aluco* | Strigidae | adult | WRC1 | trauma | mainly mammals | Sedentary | N |  |  |  | N |
| RAP25 | *Strix aluco* | Strigidae | young | WRC1 | trauma | mainly mammals | Sedentary | N |  |  |  | N |
| RAP29 | *Strix aluco* | Strigidae | adult | WRC2 | trauma | mainly mammals | Sedentary | N |  |  |  | N |
| RAP42 | *Strix aluco* | Strigidae | adult | WRC2 | other causes | mainly mammals | Sedentary | 26.472 | N | N | Type II | N |
| ^*^ N: negative results; n.d.: not determined | | | | | | | | | | | | |
